# Serotonin depletion causes valproate-responsive manic-like condition and increased hippocampal neuroplasticity that are reversed by stress

**DOI:** 10.1038/s41598-018-30291-2

**Published:** 2018-08-07

**Authors:** Giacomo Maddaloni, Sara Migliarini, Francesco Napolitano, Andrea Giorgi, Serena Nazzi, Daniele Biasci, Alessia De Felice, Marta Gritti, Anna Cavaccini, Alberto Galbusera, Sara Franceschi, Francesca Lessi, Marco La Ferla, Paolo Aretini, Chiara Maria Mazzanti, Raffaella Tonini, Alessandro Gozzi, Alessandro Usiello, Massimo Pasqualetti

**Affiliations:** 1 Department of Biology, Unit of Cell and Developmental Biology, University of Pisa, 56127 Pisa, Italy; 2 Ceinge Biotecnologie Avanzate, 80145 Naples, Italy; 3 Department of Molecular Medicine and Medical Biotechnology, University of Naples “Federico II”, Naples, Italy; 4 Functional Neuroimaging Laboratory, Istituto Italiano di Tecnologia, Center for Neuroscience and Cognitive Systems @ UniTn, 38068 Rovereto, Italy; 5 Statistics and Computational Biology Group, Cancer Research UK Cambridge Institute, Li Ka Shing Centre, Cambridge, UK; 6 Neuromodulation of Cortical and Subcortical Circuits Laboratory, Neuroscience and Brain Technologies Department, Istituto Italiano di Tecnologia, 16163 Genova, Italy; 7 Fondazione Pisana per la Scienza, 56100 Pisa, Italy; 8 Department of Environmental, Biological and Pharmaceutical Sciences and Technologies, University of Campania, Luigi Vanvitelli, Caserta, Italy; 9 Present Address: Harvard Medical School, Department of Genetics, Boston, 02115 MA USA; 10 Present Address: Emory University, Department of Physiology, Atlanta, 30307 GA USA

## Abstract

Abnormal hippocampal neural plasticity has been implicated in behavioural abnormalities and complex neuropsychiatric conditions, including bipolar disorder (BD). However, the determinants of this neural alteration remain unknown. This work tests the hypothesis that the neurotransmitter serotonin (5-HT) is a key determinant of hippocampal neuroplasticity, and its absence leads to maladaptive behaviour relevant for BD. Depletion of brain 5-HT in *Tph2* mutant mice resulted in reduced behavioural despair, reduced anxiety, marked aggression and lower habituation in novel environments, reminiscent of bipolar-associated manic behaviour. Treatment with valproate produced a substantial improvement of the mania-like behavioural phenotypes displayed by *Tph2* mutants. Brain-wide fMRI mapping in mutants revealed functional hippocampal hyperactivity in which we also observed dramatically increased neuroplasticity. Importantly, remarkable correspondence between the transcriptomic profile of the *Tph2* mutant hippocampus and neurons from bipolar disorder patients was observed. Chronic stress reversed the emotional phenotype and the hippocampal transcriptional landscape of *Tph2* mutants. These changes were associated with inappropriate activation of transcriptional adaptive response to stress as assessed by gene set enrichment analyses in the hippocampus of *Tph2* mutant mice. These findings delineate 5-HT as a critical determinant in BD associated maladaptive emotional responses and aberrant hippocampal neuroplasticity, and support the use of *Tph2*−/− mice as a new research tool for mechanistic and therapeutic research in bipolar disorder.

## Introduction

The ability of nerve cells to reshape and form new connections in response to exogenous stimuli is a process commonly referred to as neuroplasticity. This represents an essential property of the nervous system to adapt to changes in the external milieu. Among different brain regions, changes in hippocampal neuroplasticity appear to be critical in mood regulation and response to changing environment^1–3^. In particular, growing evidence indicates that adaptive mechanisms to stressful circumstances require hippocampal neuroplasticity, which is necessary to process new information coming from the environment and to organize an appropriate behavioural response^4,5^. Accordingly, abnormal hippocampal neuroplasticity has been linked to the maladaptive emotional response observed in stress-related neuropsychiatric disorders^6^. This has been extensively demonstrated in terms of reduced neurotrophic support and dampened adult neurogenesis in *post-mortem* depressed patients^7–10^, as well as in animal models of depression, which also displayed dendritic atrophy and spine loss^11,12^. Conversely, much less is known regarding the pathophysiology of bipolar disorder (BD). Recently, hippocampal hyperactivity and increased plasticity have been reported in hippocampal-like neurons derived from BD patients and in animal models of mania opening the possibility of a direct involvement of the hippocampus^13–15^. However, several questions remain unanswered. For example, the determinants and relevance of such hippocampal hyperactivity and increased plasticity remain elusive. Moreover, it is unclear whether these abnormalities are mood-phase dependent or also apply for the depressive phase of the disease.

The neurotransmitter serotonin (5-HT) is released via dense long-range projections throughout the hippocampus^16^, where it has been proposed as a pivotal player in neuroplasticity in both normal and neuropsychiatric conditions^17^. The administration of drugs increasing serotonergic signalling (e.g. Selective Serotonin Reuptake Inhibitors, SSRIs) normalizes several hippocampal neuroplasticity abnormalities, including neurotrophin levels and adult neurogenesis, as well as maladaptive behavioural responses. These effects have been reproduced in both depressed patients and animal models of depression^7,9,18,19^, leading to the classic view according to which 5-HT signalling promotes hippocampal plasticity and elevates mood-related behaviours. However, the generation of 5-HT deficient animal models challenges this view. Indeed, *Tph2* mutant mice lacking 5-HT show a paradoxical increase in hippocampal neuroplasticity^20–22^ as well as signs of markedly reduced behavioural despair and hyperactivity^23^. These observations suggest that severely impaired 5-HT transmission could lead to a maladaptive increase in synaptic plasticity and dysphoric behavioural conditions that could underlie manic-like states. According to this hypothesis, manic-states would therefore reflect a reduced serotonergic tone impinging on hippocampal plasticity.

To test this hypothesis, here we carried out behavioural experiments, *in vivo* neuroimaging, hippocampal RNA-seq, neuroanatomical, electrophysiological and biochemical analyses as well as environmental manipulations in *Tph2* mutant mice^24^. Our findings reveal that impaired 5-HT neurotransmission is a critical determinant of maladaptive behaviour and aberrant hippocampal neuroplasticity relevant for bipolar disorder.

## Methods

### Animals

*Tph2*−/− mice^24^ and control *Tph2*+/+ male littermates were housed in standard Plexiglas cages at constant temperature/humidity (22 ± 1 °C, 50–60%) and maintained on a 12/12 h light/dark cycle, with food and water *ad libitum*. All animals used in each experiment were on a C57BL/6 genetic background. Experimental protocols were conducted in accordance with the Ethic Committee of the University of Pisa and approved by the Veterinary Department of the Italian Ministry of Health.

### Experimental design and statistical analysis

*Tph2*−/− (KO) and control *Tph2*+/+ (WT) mice were individually housed after weaning, due to their high aggressive behaviour. For each behavioural test, independent cohorts were used to avoid potential confounding effects due to behavioural tests (Forced Swim Test WT n = 13, KO n = 12; Tail Suspension Test WT n = 10, KO n = 9; Novelty-Suppressed Feeding WT n = 14, KO n = 11; Neutral Arena Aggression Test n = 12). For locomotion analysis (Home Cage Locomotion n = 11), 5-minutes interval were scored for both square entries and rearings and the difference between mutants and controls were calculated for each time point separately. Independent cohorts of 10–15 weeks old male mice were used for functional Magnetic Resonance Imaging (n = 10), RNA-seq experiment (n = 3), BDNF protein level measurements (n = 8), electrophysiological recordings (WT n = 8; KO n = 9), dendritic spine analysis (WT n = 7; KO n = 8). FST experiment after unpredictable Chronic Mild Stress (uCMS) protocol was performed on independent cohorts composed by KO (n = 18) and control WT (n = 21) subjected to uCMS and KO (n = 17) and WT (n = 20) left undisturbed in their home cages. Immediately after the FST, the hippocampus of these animals was dissected and used for either BDNF/TrkB protein level measurement (WT n = 9, KO n = 8, WT-S n = 10, KO-S n = 9) or RNA-seq (n = 3). TST experiment after unpredictable Chronic Mild Stress (uCMS) protocol was performed on independent cohorts composed by KO (n = 14) and control WT (n = 15) subjected to uCMS and KO (n = 12) and WT (n = 10) left undisturbed in their home cages.

All values are expressed as mean ± s.e.m. unless stated otherwise. One- or two-way ANOVA tests with Fisher’s post hoc tests were used. For electrophysiological recordings, data were analysed by one-way repeated measures ANOVA (RM1W) for comparisons within a group; post-hoc analysis (Tukey’s) was performed only when ANOVA yielded a significant main effect. Two groups were tested for statistical significance using a Student’s *t* test. Statistically significant differences were considered at p < 0.05. Statistical analysis was performed using Statview 5.0.1 and GraphPad Prism 6.

### Behavioural testing

All behavioural procedures were performed following standard protocols during the light phase of the light/dark cycle (11:00–13:00 h). Depression-like behaviours were assessed in the Forced Swim Test (FST) and Tail Suspension Test (TST) that were performed following standard protocols. Briefly, in the FST mice were placed in a 5L Plexiglas Beaker containing 4L of 26 °C water and video-recorded for 6 min. Minutes from 2 to 6 were analysed for immobility time. In the TST, mice were hanged by their tail from a bar 50 cm from the ground with a piece of autoclave tape and were recorded in a 6 min session. Minutes from 2 to 6 were analysed for immobility time. For both tests, immobility was considered as absence of any active movement of the paws. Anxiety-like behaviour was analysed in the Novelty-Suppressed Feeding (NSF). Food was removed from the cages of mice 24 h before testing. The next day, mice were placed for 10 min in a bright white arena (38 × 35 × 20 cm) without bedding with a food pellet at the centre. Mice were video-recorded and latency to feed was assessed offline. After the test, to avoid confounding effects of feeding behaviour on anxiety, hunger was measured by weighting before and after 5 min a single pellet of food placed in the home cage. Aggressive behaviours were measured in a Neutral Arena Aggression Test (NAAT). Two mice of the same genotype were placed in a novel standard cage (42.5 × 26.5 × 18.5 cm) with bedding and video-recorded for 10 min. Lateral threats and clinch attacks were considered as sign of aggression. Latency to the first attack, attack duration and number of attacks in 10 min were measured. Locomotor habituation to novelty was measured in the Novel Home-Cage (NHC) paradigm. Briefly, mice were individually placed into a novel standard cage (42.5 × 26.5 × 18.5 cm) with bedding and video-recorded for 60 min. The area of the cage was virtually subdivided in squares and the times the testing mouse crossed one of the grid lines with all four paws (i.e. square entry) was scored, as well as the times it stood on its hind legs (i.e. vertical activity, also known as rearing). Hedonic behaviour was assessed by sucrose preference test. Briefly, single-housed male mice were habituated to the presence of two bottles containing water for two days. On day 0, bottles were replaced by one containing water and the other containing 1% sucrose solution in drinking water. The two bottles were switched every 12 hours to reduce side bias and weighed every 24 hours.

### Valproate treatment

Valproate sub-chronic treatment was performed according to Flaisher-Grinberg and Einat^25^. Briefly, Valproate (Sigma) was dissolved in saline solution to obtain a dose of 100 mg/kg in 10 ml/kg injection volume. Mice were injected intraperitoneally twice a day (12 h interval) for two consecutive days. On the third day, mice received the last injection 30 min before the behavioural test. Control mice received an equivalent volume of saline solution.

### *In vivo* functional Magnetic Resonance Imaging (fMRI)

#### Animal preparation

Magnetic Resonance Imaging experiments were performed on adult Tph2+/+ (n = 10) and Tph2−/− (n = 10) littermate male mice. Briefly, mice were anaesthetized with isoflurane (5%), intubated and artificially ventilated. The left femoral artery was cannulated for contrast agent administration, continuous blood pressure monitoring and blood sampling. At the end of surgery, isoflurane was discontinued and substituted with halothane. Experiments were carried out at a maintenance anesthesia level of 0.8%. Arterial blood gases (paCO_2_ and paO_2_) were measured at the end of the functional time series. The values recorded were 16 ± 4 mmHg (paCO_2_), 287 ± 95 mmHg (paO_2_) and 17 ± 4 mmHg (paCO2), 272 ± 86 mmHg (paO_2_) for Tph2−/− and control, respectively. No significant inter-group difference in paCO_2_ or (paO_2_) levels was observed between groups (p > 0.65, Student’s t test). Functional data acquisition commenced 30 min after isoflurane cessation.

#### Image Data Acquisition

All experiments were performed using a 7.0 Tesla MRI scanner (Bruker Biospin, Milan). Transmission and reception were achieved using a 72 mm birdcage transmit coil and a custom-built saddle-shaped solenoid coil for signal reception. Shimming was performed on a 6 mm × 6 mm × 6 mm region, using a FASTMAP protocol. For each session, high-resolution anatomical images were acquired with a fast spin echo sequence (RARE) with the following parameters: repetition time (TR)/echo time (TE) 3550/40 ms, matrix 192 × 192, field of view 2 × 2 cm^2^, 28 coronal slices, slice thickness 0.50 mm. Co-centered Cerebral Blood Volume (CBV) weighted fMRI times series were acquired using a Fast Low-Angle Shot (FLASH) MRI sequence with the following imaging parameters: FLASH TReff = 283.023 ms, TE = 3.1 ms, α = 30°; FOV 2 × 2 cm^2^, 156 × 156 × 500 µm resolution, dt = 60 s, Nr = 60, corresponding to 60 min total acquisition time. Images were sensitized to reflect alterations in CBV by injecting 5 µl/g of superparamagnetic iron oxide (Molday Ion, Biopal) intra-arterially after 5 baseline images.

#### Basal CBV mapping

To calculate basal CBV (bCBV), CBV-weighted time series were spatially normalized to a study-based anatomical template, and signal intensity was converted into basal cerebral blood volume (bCBV(t)) pixel-wise. bCBV time-series were calculated over a 5 minute time-window starting 15 min after contrast agent injection. Voxel-wise group statistics was carried out using FSL using multi-level Bayesian inference and a T threshold >2.1, and corrected cluster significance threshold of p = 0.01.

### RNA extraction and whole transcriptome RNA analysis

Whole hippocampal tissue from n = 3 mice was rapidly dissected and quickly frozen in liquid nitrogen. Total RNA was extracted using the automated Maxwell 16 LEV RNA FFPE Purification Kit with the Maxwell 16 Instrument (Promega, Madison, WI, USA). We followed the manufacturer’s instructions protocol starting from the Lysis Buffer and Proteinase K step excluding the Mineral Oil procedure. Hippocampus tissue was homogenized in Lysis Buffer using a pestle.

RNA-seq was performed using NextSeq. 500 (Illumina, San Diego, CA, US) for Next Generation Sequencing. The library was prepared following the protocol TruSeq Stranded mRNA LT kit (Illumina). Libraries were quantified using Qubit 2.0 Fluorometer (Invitrogen, Life Technologies, Grand Island, NY) and the size profile was analyzed on the 2200 TapeStation instrument (Agilent Technologies, Santa Clara, CA).

Raw data were converted to FASTQ format using bcl2fastq (Illumina). We used the FastQC quality control tool (http://www.bioinformatics.babraham.ac.uk/projects/fastqc/) to perform quality assessment. In addition, we evaluated raw data contamination from different organisms (bacteria, fungi, virus) by applying FastqScreen (http://www.bioinformatics.babraham.ac.uk/projects/fastq_screen/). RNA-Seq reads were aligned to the mouse genome (mm10; UCSC) with STAR aligner 2.5.1 (https://github.com/alexdobin/STAR). Differential expression between conditions was calculated using Cuffdiff (http://cole-trapnell-lab.github.io/cufflinks/cuffdiff/). All RNA-Seq analyses were performed in the cloud using the Seven Bridges Genomics platform (www.sbgenomics.com). Gene Set Enrichment Analysis was performed using GSEA (http://software.broadinstitute.org/gsea/). Hierarchical gene clustering on differentially expressed genes was performed using Bioconductor ctc package on R software 3.0.1 (https://www.bioconductor.org/packages/release/bioc/html/ctc.html).

### Electrophysiological recordings

Extracellular recordings of field postsynaptic potentials (fPSP) were obtained in the CA1 stratum radiatum, using glass micropipettes filled with artificial Cerebral Spinal Fluid (aCSF). Stimuli (50–160 μA, 50 μs) to excite Shaffer collaterals were delivered through a bipolar twisted tungsten electrode placed 400 μm from the recording electrode. Long-Term Potentiation (LTP) was induced using the following theta burst stimulation protocol (TBS): 10 trains (4 pulses at 100 Hz) at 5 Hz, repeated twice with a 2-min interval. The magnitude of LTP was evaluated by comparing the fPSP normalized slopes from the last 5 min of baseline recordings with those 40–50 min after TBS.

For patch-clamp recordings, whole-cell recordings were made under direct IR-DIC (infrared-differential interference contrast) visualization of neurons in the hippocampal CA1 stratum pyramidale region. Excitatory postsynaptic currents (EPSCs) were evoked in the presence of the GABA_A_ receptor antagonist gabazine (10 μM) by stimulation of stratum radiatum by using a theta glass electrode (20 µsc–80 µsc, 0.02 mA–0.1 mA) connected to a constant-current isolation unit (Digitimer LTD, Model DS3) and acquired every 5 seconds. The glass of theta electrode, composed by two isolated channels, was pulled to produce the tip for microstimulation and each channel of the glass tip was filled with a normal aCSF used during recordings. Voltage clamp experiments were performed on CA1 pyramidal neurons using borosilicate patch pipettes (3–4 MΩ) filled with a solution containing (in mM): 135 CsMeSO_3_, 5 CsCl, 5 NaCl, 2 MgCl_2_, 0.1 EGTA, 10 HEPES, 0.05 CaCl_2_, 2 Na2-ATP, 0.4 Na_3_-GTP (pH 7.3, 280–290 mOsm/kg). Each CA1 pyramidal neuron was voltage-clamped at −70 mV and at +40 mV to evoke AMPA and NMDA receptor-mediated EPSCs respectively. AMPA and NMDA EPSCs were recorded before and after blocking AMPA mediated currents by bath applying 20 µM NBQX disodium salt. Access resistance was monitored throughout the experiment. Signals were sampled at 10 kHz filtered at 2.8 kHz. Series resistance (range 15–20 MΩ) was monitored at regular intervals throughout the recording and presented minimal variations (≤20%) in the analyzed cells. Data are reported without corrections for liquid junction potentials. Data were acquired using a Multiclamp 700B amplifier controlled by pClamp 10 software (Molecular Device), with a Digidata 1322 (Molecular Device). AMPA/NMDA ratio of each neuron was calculated as the ratio between AMPA EPSC peak amplitude (pA) of the subtracted current and the NMDA EPSC peak amplitude (pA).

### Immunohistochemistry and 3D modelling analysis

Animals were perfused transcardially with 4% paraformaldehyde (PFA), brains were dissected, post-fixed o/n at 4 °C and coronal sections (50 μm thick) were obtained with a vibratome (Leica Microsystems). Immunohistochemistry was performed following standard protocols. Briefly, free-floating sections were permeabilized with 0.5% Triton-X100 (Sigma) in PBS. Sections were then blocked in 5% horse serum (Gibco, Life Technologies), 0.5% Triton-X100 in PBS for 1 h followed by overnight incubation with the primary antibody (rabbit anti-GFP antibody, 1:2000, Molecular Probes) at 4 °C. After six washes with 0.5% Triton-X100 in PBS, sections were incubated overnight with the secondary antibody (Rhodamine Red-X goat anti-rabbit IgG, 1:500, Molecular Probes) at 4 °C. After three washes with 0.5% Triton-X100 in PBS, section were incubated with DAPI (0.1 μg/ml, Sigma), washed three times with PBS and then mounted onto glass slides and coverslipped with Aqua Poly/Mount (PolyScience).

For dendritic spine analysis, adult Tph2−/− mice and control mice carrying the Thy1-YFP-M allele were processed for immunohistochemistry using anti-GFP antibody and a rhodamine-conjugated secondary antibody to avoid interference of endogenous YFP fluorescence. A number of 6 confocal fields (35 Z-steps at 0.15μ interval) on consecutive coronal sections of both CA1 and CA3 fields in the dorsal hippocampus were imaged using a Nikon A1 confocal microscope equipped with a 60x PlanApo oil objective at 1024 × 1024 pixel resolution. Images were analyzed using the Filament Tracer semi-automated method for dendrites and spine properties quantifications (Imaris 7.2.3, Bitplane). For each animal, 20 to 30 dendrites in the apical region were reconstructed for each hippocampal field.

### Unpredictable Chronic Mild Stress (uCMS)

uCMS paradigm was modified from Tye *et al*.^26^. Age-matched Tph2−/− and control mice were randomly subdivided in the uCMS and control group. Control mice of both genotypes were housed in standard conditions. uCMS protocol consisted in two stressors per day (one during the day, one during the night) for 8 weeks. Cage tilt on a 45° angle for 16 h, food deprivation for 6 h, white noise (http://www.simplynoise.com) for 16 h, continuous illumination for 36 h, 3 h darkness during the light cycle, continuous darkness for 36 h, water deprivation for 6 h, wet bedding (150 mL water into sawdust bedding) for 16 h, rat feces exposure in the cage for 16 h, cage switching between mice, restraint stress in 50 mL tube for 2 h, overcrowded housing for 3 h were the stressors randomly applied to be unpredictable for mice. Except for overcrowding, as well as for water and food deprivation sessions, water and food were available ad libitum. Tph2−/− and WT mice from uCMS and control groups were behaviorally tested with the Forced Swim Test 24 h after the last uCMS session, and then immediately sacrificed. Brains were rapidly removed, hippocampal tissue dissected and quickly frozen in liquid nitrogen for Western blot and RNA-seq analyses.

### Western Blotting

Biochemical studies were performed as reported in Napolitano *et al*.^27^. The hippocampi were sonicated in a lysis buffer (320 mM sucrose, 50 mM Tris HCl pH 7.5, 50 mM NaCl, 1% Triton X-100, 5 mM β-glycerol phosphate, 1 mM Na3VO4, 5 mM NaF, protease inhibitor cocktail) and incubated on ice for 30 min. Samples were spin at 12,000 g × 10 min and the supernatant transferred to fresh microfuge tube. Aliquots of the homogenate were used for protein determination using Bio-Rad Protein Assay kit (Bio-Rad, Hercules, CA). Equal amounts of total proteins (30 μg) for each sample were loaded onto 15% (for BDNF detection) or 10% (for TrkB detection) polyacrylamide gels. Proteins were separated by SDS-PAGE and transferred overnight to membranes (PVDF; Amersham Pharmacia Biotech, Uppsala, Sweden). The membranes were immunoblotted overnight using selective antibodies against BDNF and TrkB (each diluted 1:1000, Santa Cruz Biotechnology). Both BDNF and TrkB optical density values were normalized using antibodies against GAPDH (1:1000, Santa Cruz Biotechnology) and α-Tubulin (1:50000, Sigma Aldrich), respectively. Blots were then incubated in horseradish peroxidase-conjugated secondary antibodies and target proteins visualized by ECL detection (Pierce, Rockford, IL), followed by quantification by Quantity One software (Biorad). Normalized values were averaged and used for statistical analysis performed by two-way ANOVA followed by post-hoc comparison, when required.

## Results

### *Tph2* knockout mice show mania-like behaviours and increased functional activity in the hippocampus

**Figure 1:**
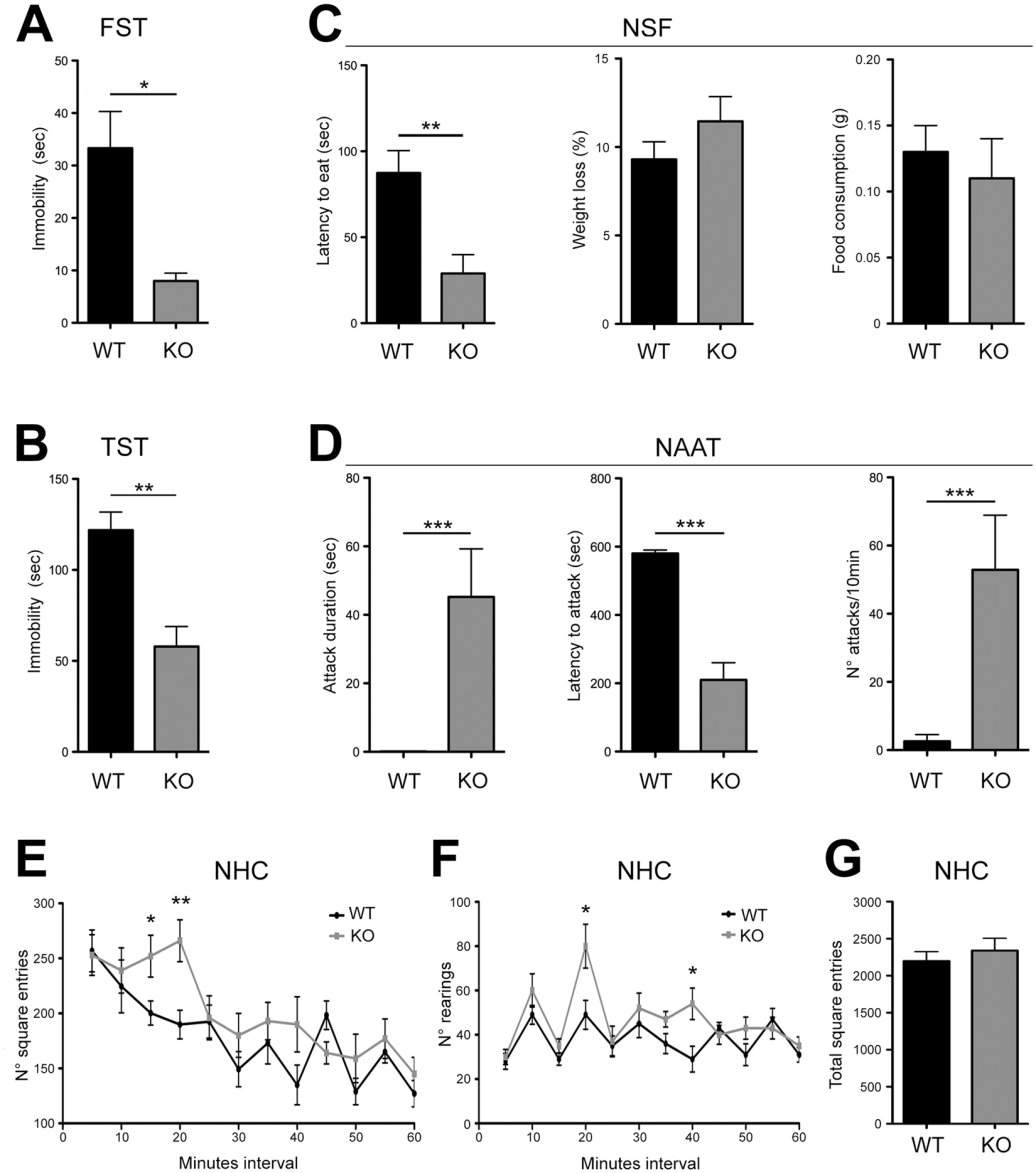
KO mice show mania-like behaviours. (**A**) Immobility time in the FST (WT n = 13, KO n = 12; one-way ANOVA, F_1,23_ = 4.317, p = 0.0491) and (**B**) TST (WT n = 10, KO n = 9; one-way ANOVA, F_1,17_ = 8.099, p = 0.0112). (**C**) Latency to eat, weight loss and food consumption in the NSF (WT n = 14, KO n = 11; one-way ANOVA, F_1,23_ = 8.192, p = 0.0088). (**D**) Attack duration (n = 12; one-way ANOVA, duration: F_1,22_ = 13.073, p = 0.0012), latency to the first attack (n = 12; one-way ANOVA, F_1,22_ = 49.018, p = 0.000095) and attack frequency (n = 12; one-way ANOVA, F_1,22_ = 15.266, p = 0.0014) in the NAAT. (**E**) Number of square entries (n = 11, one-way ANOVA, square entries: F_1,20_ = 4.519, p = 0.0469 at 15 min; F_1,20_ = 9.595, p = 0.0055 at 20 min) and (**F**) number of rearings (one-way ANOVA, rearings: F_1,20_ = 6.182, 0.021 at 20 min; F_1,20_ = 7.539, p = 0.0223 at 40 min) in NHC paradigm, respectively. (**G**) Total square entries in the NHC paradigm. Data are expressed as mean ± s.e.m., *p < 0.05, **p < 0.01, ***p < 0.001. FST, Forced Swim Test; TST, Tail Suspension Test; NSF, Novelty-Suppressed Feeding; NAAT, Neutral Arena Aggression Test; NHC, Novel Home-Cage. n indicates biological replicates.

To link 5-HT deficiency to the onset of manic-like phenotypes, we first performed a battery of behavioural tests on *Tph2*−/− mice (KO) and *Tph2*+/+ littermates (WT). KO mice displayed markedly reduced immobility in both the Forced Swim Test (FST, Fig. 1A) and the Tail Suspension Test (TST, Fig. 1B) as well as reduced latency to feed in a novel environment in the Novelty-Suppressed Feeding Test (NSF, Fig. 1C), which were maintained with ageing (Supplementary Fig. S1). In the Neutral Arena Aggression Test (NAAT), KO mice showed escalated aggression (Fig. 1D). Mutants exhibited a more intense exploratory behaviour in a Novel Home Cage paradigm (NHC, Fig. 1E) and increased number of rearings relative to controls (Fig. 1F) with no significant differences in total locomotion (Fig. 1G). No genotype effect was observed in the sucrose preference test (Supplementary Fig. S2). Altogether, behavioural testing revealed reduced depression- and anxiety-like behaviours, increased risk-taking, unusual aggression as well as slower locomotor habituation to novelty in KO mice, which together are considered mania-like behavioural phenotypes in rodents^28,29^, modelling traits of the BD manic phase.

**Figure 2:**
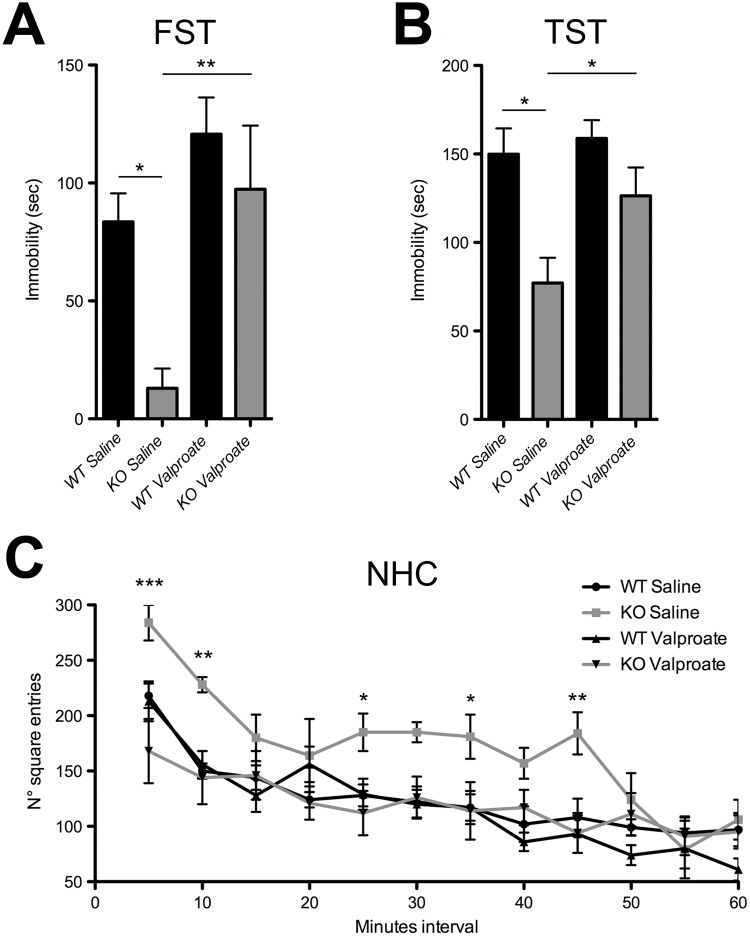
Sub-chronic treatment with valproate rescued the mania-like phenotype in KO mice. (**A**) Immobility time in the FST (WT Saline n = 9, KO Saline n = 7, WT Valproate n = 9, KO Valproate n = 7; two-way ANOVA followed by Fisher’s post-hoc tests, effect of treatment F_1,29_ = 12.201, p = 0.016; effect of genotype F_1,28_ = 7.28 p = 0.015; KO Saline vs WT Saline p = 0.0002, KO Saline vs KO Valproate p = 0.0027) and (**B**) TST (WT Saline n = 8, KO Saline n = 8, WT Valproate n = 5, KO Valproate n = 8; two-way ANOVA followed by Fisher’s post-hoc tests, effect of genotype F_1,25_ = 11.63, p = 0.0022; KO Saline vs WT Saline p = 0.0018, KO Saline vs KO Valproate p = 0.0208). (**C**) Number of square entries (WT Saline n = 9, KO Saline n = 6, WT Valproate n = 9, KO Valproate n = 8; two-way ANOVA followed by Fisher’s post-hoc tests, genotype x treatment interaction F_3,28_ = 5.402, p = 0.035; KO Saline vs KO Valproate, square entries: p = 0.012 at 5 min; p = 0.0189 at 10 min; p = 0.0364 at 25 min; p = at 35 min; p = 0.0048 at 45 min) in NHC paradigm. Data are expressed as mean ± s.e.m., *p < 0.05, **p < 0.01, ***p < 0.001. FST, Forced Swim Test; TST, Tail Suspension Test; NHC, Novel Home-Cage. n indicates biological replicates.

To examine the predictive validity of *Tph2* KO mice as a novel model of bipolar mania, we tested the *in vivo* effect of valproate, a widely prescribed mood stabilizer used to treat BD patients. Interestingly, we reported that sub-chronic valproate treatment normalized the immobility time of KO mice in the FST and TST to the values shown by WT littermates (Fig. 2A,B). Moreover, valproate administration also attenuated the motor hyperactivity observed in KO mice under basal condition (Fig. 2C). Overall, these experiments indicate that the manic-related phenotypes found in KO animals are sensitive to valproate treatment.

**Figure 3:**
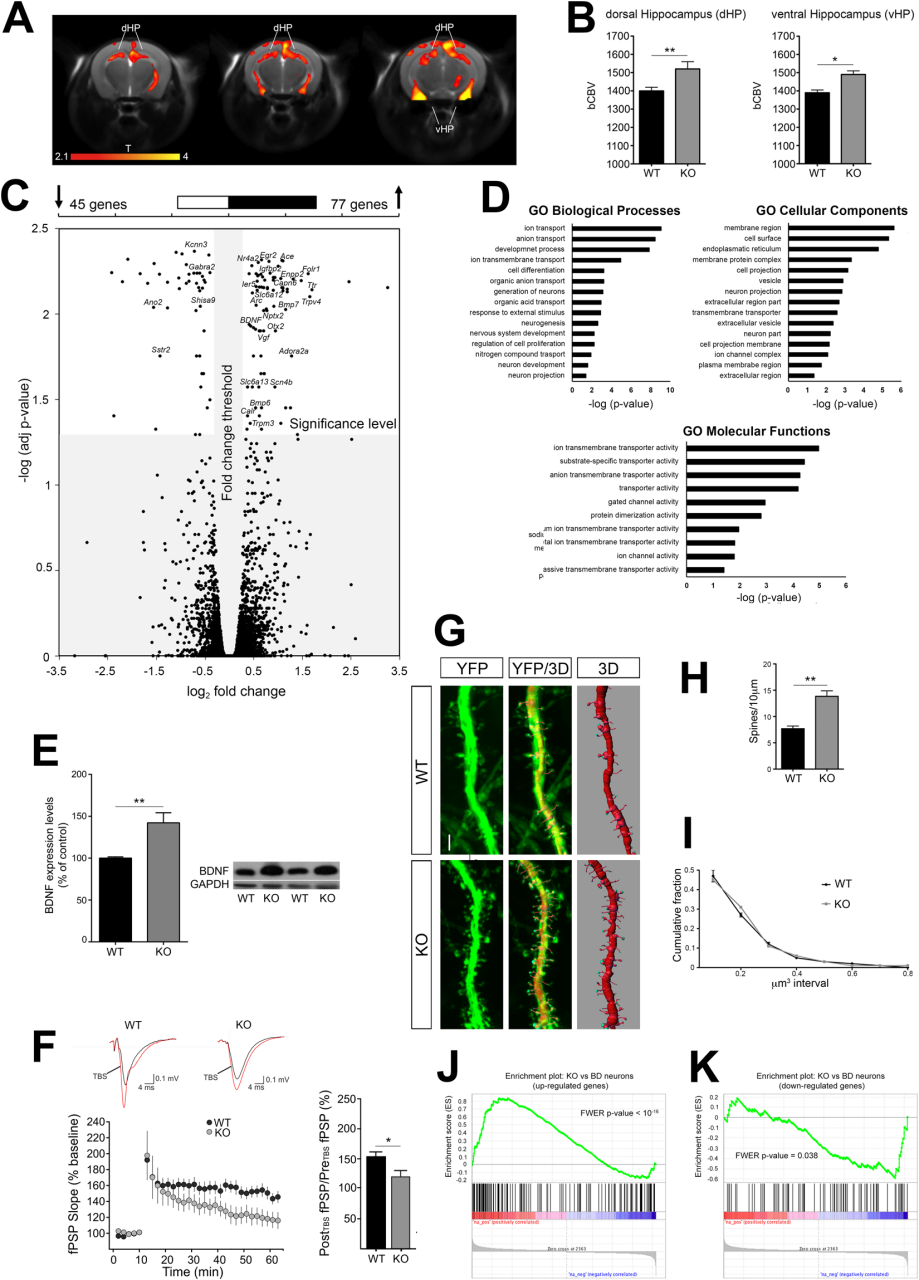
5-HT depleted mice display increased hippocampal functional activity and neuroplasticity, as well as common transcriptional abnormalities with neurons derived from BD patients. (**A**)Anatomical distribution and (**B**) quantification of brain areas exhibiting a significant increase in bCBV in KO mice with respect to control WT littermates (n = 10, one-way ANOVA: dHPC, F_1,18_ = 6.632, p = 0.0191; vHPC, F_1,18_ = 5.620, p = 0.0291; T > 2.1, corrected cluster significance p = 0.01). Foci of increased bCBV (red/orange) are superimposed onto contiguous 0.75 mm MRI coronal images. The effect has been quantifies in hippocampal areas on slice-to-slice basis. (**C**) Scatterplot of p_adj_-values versus fold-change between WT and KO mice for all genes. Grey area indicates cut-offs for significance. Upper bar: number of DE genes significantly up-regulated (right) and significantly downregulated (left) in KO mice as compared to controls. (**D**) Representative Gene Ontology categories identified from the DE genes. (**E**) Quantification and representative images of BDNF protein levels (n = 8, one-way ANOVA, F_1,14_ = 12.078, p = 0.0037) measured by Western Blotting in WT and KO mice. Images are cropped from different blots. Full length blots are displayed in Supplementary Fig. S3. Data are expressed as percentage of control ± s.e.m. **p < 0.01. (**F**) Theta burst stimulation (TBS) induces LTP at CA3-CA1 synapses of WT mice (n = 8, RM1W, F_7,30_ = 22, p < 0.0001; Tukey’s p < 0.05). In 5-HT depleted KO mice, LTP was significantly impaired (n = 9, RM1W, F_8,30_ = 7, p = 0.0111, Tukey’s p > 0.05), and reduced compared to controls (WT vs KO Student’s t test p = 0.0232). (**G**) Representative images of YFP-labeled CA3 apical dendrites and their 3D reconstruction. (**H**) Quantification of spine density (WT n = 7; KO n = 8, for spine density one-way ANOVA, F_1,13_ = 18.569, p = 0.0008) and (**I**) volume distribution in CA3 dendrites. (**J**,**K**) GSEA-calculated enrichment between the transcriptional profile of KO mice and that of neurons derived from patients with BD for (**J**) upregulated and (**K**) downregulated genes. Data are expressed as mean ± s.e.m. **p < 0.01. bCBV, basal Cerebral Blood Volume; dHPC, dorsal hippocampus; vHPC, ventral hippocampus. n indicates biological replicates. Scale bar = 2 μm.

To probe the brain-wide substrates that might underlie the observed behavioural phenotype, we measured *in vivo* basal cerebral metabolism by means of basal cerebral blood volume (bCBV) weighted functional Magnetic Resonance Imaging^30,31^ (fMRI). Interestingly, fMRI scans highlighted foci of increased metabolism that were confined to dorsal and ventral hippocampal areas of KO mice with respect to WT littermates (Fig. 3A,B).

### 5-HT depleted mice display increased hippocampal neuroplasticity and common transcriptional abnormalities with neurons derived from BD patients

We next performed total RNA sequencing in the hippocampus of KO and we identified 122 differentially expressed (DE) genes in 5-HT depleted mice relative to WT controls, with a p-value adjusted for false discovery rate (p_adj_) ≤0.05 (Fig. 3C). DE genes were enriched in the Gene Ontology (GO) categories of ion transport, neurogenesis, neuron projection, and regulation of cell proliferation (Fig. 3D and Supplementary Tables S1–S3). KOs showed increased expression of immediate-early genes (*Arc*, *Nr4a2*, *Egr2*, *Ier5*), ion channels (*Scn4b*, *Trpv4*, *Trpm3*), as well as genes involved in neuroplasticity (*BDNF*, *Nr4a2*, *Bmp6*, *Bmp7*, *Vgf*, *Enpp2*, *Igfbp2*, *Otx2*, *Calr*, *Capn6*, *Folr1*, *Ttr*, *Adora2a*, *Ace*) and neurotransmission (*Nptx2*, *Slc6a12*, *Slc6a13*; Fig. 3C). In contrast, the expression of chloride and potassium channels (*Ano2*, *Kcnn3*) as well as of other genes involved in inhibitory pathways (*Gabra2*, *Sstr2*, *Shisa9*) was decreased (Fig. 3C).

Among the other DE genes identified, Brain-Derived Neurotrophic Factor (BDNF) has been shown to play a pivotal role in both synaptic potentiation and structural hippocampal plasticity^32^. In line with RNA-seq results and previous reports^24,33^, we found increased BDNF protein levels in the hippocampus of KO mice (Figs 3E and S3). We induced Long-Term Potentiation (LTP) in hippocampal slices by θ-burst stimulation (TBS) to investigate the BDNF-mediated hippocampal synaptic plasticity^34,35^. TBS-LTP was severely impaired in KO animals as compared to WT (WT: 154 ± 8% of baseline, KO: 120 ± 11% of baseline; Fig. 3F), suggesting that CA3-CA1 synapses may be potentiated and could not be further experimentally manipulated. This hypothesis was corroborated by the AMPA/NMDA ratio showing a trend towards an increase in KO vs WT animals (Supplementary Fig. S4). We next investigated dendritic spine density and morphology along apical dendrites of hippocampal pyramidal neurons. Results showed in a dramatic increase of spine density in CA3 of KO animals with respect to control littermates (Fig. 3G,H), and a trend for a similar effect in CA1 (Supplementary Fig. S5). The distribution of spine volume was comparable in the two genotypes (Figs 3I and S5). Collectively, these data demonstrate that lack of serotonin leads to selective hippocampal hyperactivity together with increased synaptic and structural plasticity.

Hippocampal-like neurons in human BD patients exhibit hyper-excitability and increased expression of neurotransmission-related genes^13^. To establish a mechanistic link between our finding and human BD, we used Gene Set Enrichment Analysis^36^ (GSEA) to compare transcriptional differences between BD neurons^13^ (GSE58933) and *Tph2* mutants. Results showed statistically significant enrichment of the KO mouse transcriptional signature as compared to that of BD neurons. Genes upregulated in KO mice compared to WT were also upregulated in neurons derived from BD patients compared to healthy controls (Bonferroni corrected p < 10^−16^; Fig. 3J), and genes downregulated in KO mice were also downregulated in BD neurons (Bonferroni corrected p = 0.038; Fig. 3K). These correspondences suggest a possible mechanistic relevance of our findings for human BD states.

### Chronic stress reverses the emotional phenotype and the hippocampal transcriptional signature in Tph2 KO mice

**Figure 4:**
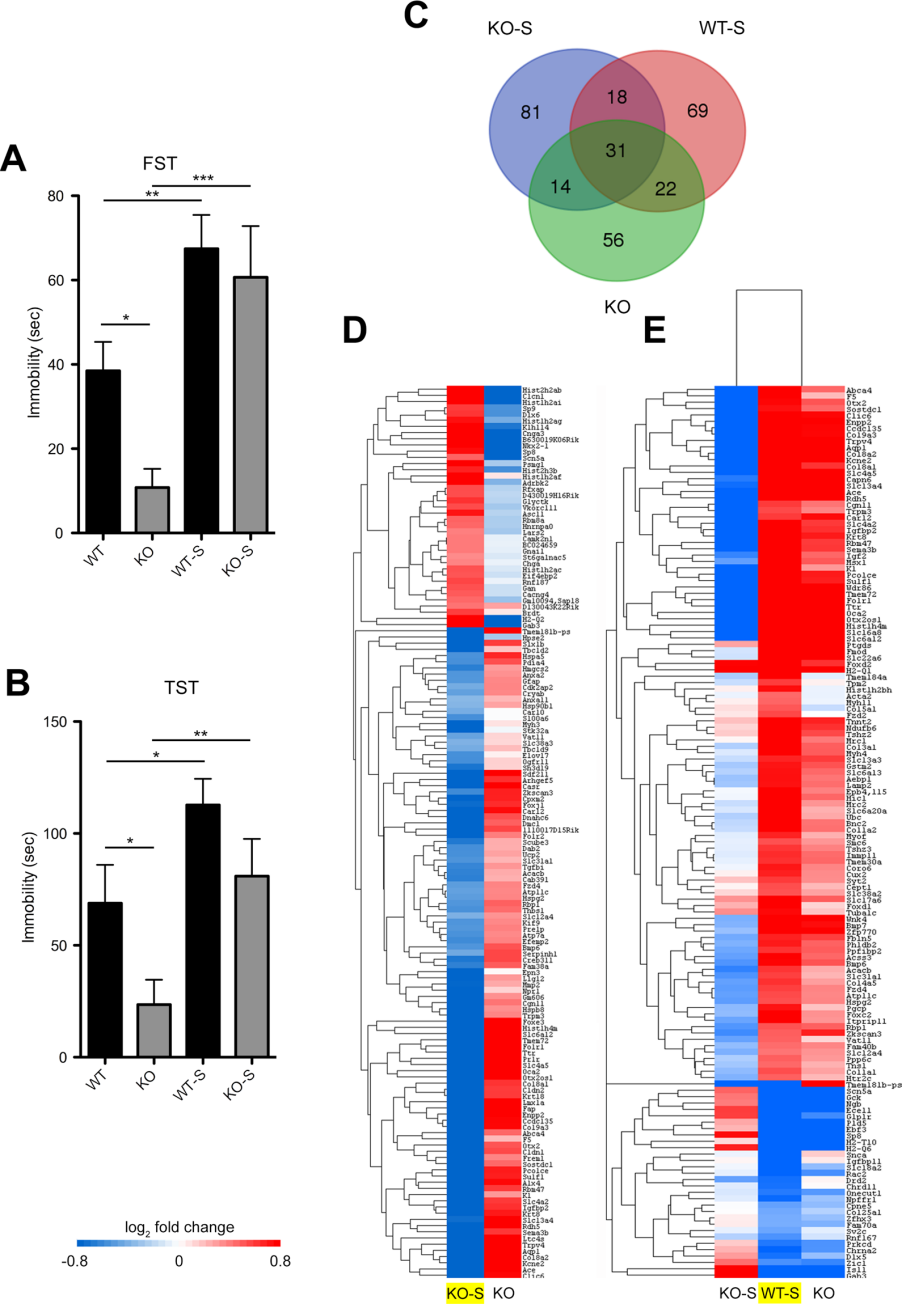
Chronic stress reverses the emotional phenotype and the hippocampal transcriptional signature in Tph2 KO mice. (**A**) Immobility time in the FST (WT n = 20, KO n = 18, WT-S n = 21, KO-S n = 17; two-way ANOVA followed by Fisher’s post-hoc tests, effect of uCMS F_3,72_ = 14.907, p = 0.0002. No genotype x uCMS interaction; KO-S vs KO F_1,31_ = 12.825, p = 0.0002) and (**B**) TST (WT n = 10, KO n = 12; WT-S n = 15, KO-S n = 14; two-way ANOVA followed by Fisher’s post-hoc tests, effect of uCMS F_3,72_ = 12.282, p = 0.001. No genotype x uCMS interaction; KO-S vs KO F_1,24_ = 7,646, p = 0.0108) of WT-S and KO-S mice and their respective controls. (**C**) Overlapping distribution of DE genes in a Venn diagram across the three comparisons (KO-S = KO-S vs KO; WT-S = WT-S vs WT; KO = KO vs WT). (**D**) Heat map showing how DE genes in the KO-S/KO comparison (in yellow) are regulated in the KO/WT comparison. (**E**) Heat map showing how DE genes in the WT-S/WT (in yellow) are regulated in the KO-S/KO and KO/WT comparisons. n indicates biological replicates. FST, Forced Swim Test; TST, Tail Suspension Test; uCMS, unpredictable Chronic Mild Stress.

In BD patients abnormal mood transitions can be triggered by stress^37,38^. To assess whether the manic-like behaviour of 5-HT depleted mice is similarly reversed by stress, we exposed WT and KO mice (namely WT-S and KO-S, respectively) to an eight-weeks unpredictable Chronic Mild Stress (uCMS) protocol. We then performed FST (Fig. 4A) and TST (Fig. 4B) which showed in KO mice a clear reversion of the manic-like behaviour following uCMS. Interestingly, we observed no significant difference in the aggression levels of KO-S animals following uCMS (Supplementary Fig. S6), in keeping with the evidence that BD patients display overt aggression independently of the polarity of the mood episode^39^.

To characterize the transcriptional networks underpinning the depressive-like state of KO animals, we performed RNA sequencing on the hippocampus of mice subjected to uCMS. Results showed that stress significantly affected the expression of 144 genes in KO-S mice as compared to KO counterparts (Fig. 4C). DE genes were enriched in the GO categories of ion transport, system development, and positive regulation of biological processes among others (Supplementary Tables S4–S6). Remarkably, 45 of them (e.g. *Otx2*, *Ttr*, *Folr1*, *Enpp2* and *Trpv4*) resulted to be DE in the KO/WT comparison but showing a reversed gene expression. This change highlighted a lowering of neuroplasticity factors when KO mice switch from manic- to depressive-like behaviours (Fig. 4D). Further analysis revealed that, out of 140 DE genes identified in the WT-S/WT comparison (Fig. 4C,E; see also GO categories of ion transport, developmental process and extracellular matrix organization among others, detailed in Supplementary Tables S7–S9), 53 were DE genes in the KO/WT comparison, all but 1 with the same direction (Fig. 4E).

### Impaired hippocampal adaptive mechanism to stress in KO mice

**Figure 5:**
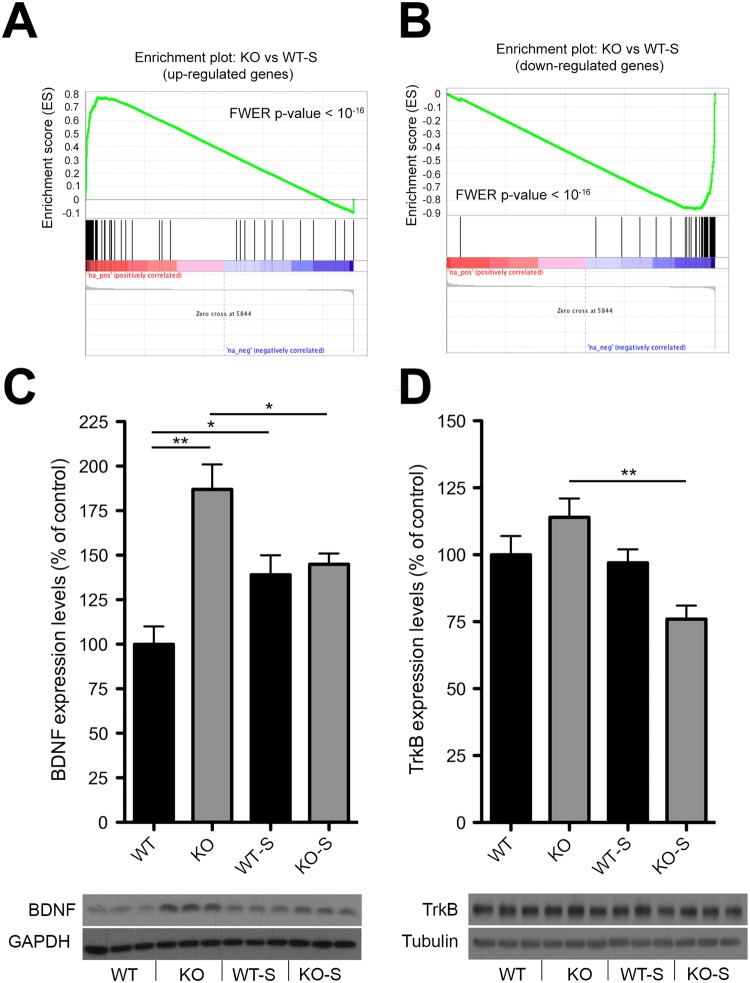
Impaired hippocampal adaptive mechanism to stress in KO mice. (**A**,**B**) GSEA-calculated enrichment score between the transcriptional profile of WT-S and KO mice. (**C**,**D**) Quantification and representative images of (**C**) BDNF (WT n = 9, KO n = 8, WT-S n = 10, KO-S n = 9, two-way ANOVA followed by Fisher’s post-hoc tests, genotype x uCMS interaction F_3,31_ = 12.066, p = 0.0015; WT vs KO F_1,15_ = 23.33, p = 0.0002; WT vs WT-S F_1,17_ = 6.744, p = 0.0188; KO vs KO-S F_1,15_ = 5.486, p = 0.0345) and (**D**) TrkB (WT n = 9, KO n = 8, WT-S n = 10, KO-S n = 9, two-way ANOVA followed by Fisher’s post-hoc tests, genotype x uCMS interaction F_3,31_ = 5.607, p = 0.0015; KO vs KO-S F_1,14_ = 15.859, p = 0.0014) protein levels measured by Western Blotting in WT-S and KO-S mice and their respective controls. Images are cropped from different blots. Full length blots are displayed in Supplementary Fig. S7. Data are expressed as mean ± s.e.m. for behavioural data, as percentage of control ± s.e.m. for biochemical data. *p < 0.05, **p < 0.01, ***p < 0.001. n indicates biological replicates.

As we detected substantial transcriptional identity between WT-S and KO mice, we next performed GSEA to quantify such similarity. Notably, we found a robust significant enrichment between the transcriptional profile of WT-S and KO mice (Bonferroni corrected p < 10^−16^ for both up- and downregulated genes; Fig. 5A,B). As we have shown that KO mouse signature was associated with hyperactivity and increased neuroplasticity, these data suggest the establishment of a transcriptional profile in WT-S mice likely promoting neuroplasticity as a stress-adaptive strategy. Interestingly, the comparison of KO-S and WT-S signature suggests that 5-HT deficiency blunts adaptive gene programs in the presence of environmental insults (Fig. 4E). To probe this hypothesis we analysed hippocampal BDNF/TrkB pathway. This pathway has been shown to be upregulated 24 h after the final stress session in rodents, implicating neuroplasticity events likely involved in adaptive mechanisms to stress^4,5,40^. In line, WT-S mice exhibited increased BDNF levels 24 h after the last uCMS session, whereas KO-S mice displayed reduced expression for both BDNF (Figs 5C and S7) and its receptor TrkB (Figs 5D and S7), corroborating the evidence that lack of 5-HT leads to an impaired mechanism of stress adaptation.

## Discussion

Here we provide evidence that congenital 5-HT deficiency results in distinct behavioural endpoints modelling bipolar-associated manic behaviour that are normalized by valproate treatment and paroxystic hippocampal functional activity. We also show a remarkable correspondence between the hippocampal transcriptomic signatures of *Tph2* KO and that observed in neurons from bipolar disorder patients. The observed abnormal transcriptomic landscape is reversed in knockout mice showing a transition from manic- to depressive-like behaviour, suggesting that hippocampal neuroplasticity is a good predictor of the mood phase experienced. These changes are plausible consequence of a maladaptive transcriptional response to stress as assessed by gene set enrichment analyses and BDNF/TrkB levels in the hippocampus of *Tph2* mutant mice.

Evidence that neuroplasticity defects and behavioural abnormalities observed in depression were rescued by 5-HT-elevating pharmacological drugs such as SSRIs, led to the hypothesis that decreased 5-HT levels underlie reduced hippocampal neuroplasticity and depressive behaviour^9,41,42^. However, the advent of refined genetic mouse models allowing 5-HT synthesis abrogation challenged this view, providing evidence of opposing 5-HT manipulations via either SSRI treatment or *Tph2* genetic ablation showed similar behavioural phenotype and neuromolecular response in several hippocampal neuroplasticity paradigms. Indeed, increased expression of BDNF has been described following SSRI administration^43,44^ or 5-HT depletion in *Tph2* knockout mice^24,33^ including the present study. Accordingly, both boosting and depleting 5-HT content results in increased adult hippocampal neurogenesis and dendritic arborisation^21,22,45^ as well as reduced behavioural despair^18,20^. However, while SSRI therapeutic effects have been observed in depressed patients and animal models of depression, increased hippocampal neuroplasticity and mood elevation have been described in 5-HT deficient mice housed in basal condition.

Guided by the observation that BD patients and animal models of mania display heightened hippocampal neuroplasticity, we hypothesized that 5-HT deficiency may trigger the onset of manic-like phenotypes. In keeping with the hypothesis, we show that *Tph2* knockout mice display reduced depression- and anxiety-like behaviours, increased risk-taking, unusual aggression as well as slower locomotor habituation to novelty in KO mice, which are often regarded as mania-like behavioural phenotypes in rodents^28,29^, modelling traits of the BD manic phase.

In humans, BD associated manic states are often misdiagnosed as attention deficit hyperactivity disorder^46^ (ADHD). Recently, the hyperactivity observed in 5-HT-depleted mouse models has been interpreted as an ADHD symptom^23^. Notably, 9–13 months old KO mice display reduced depression- (Fig. S1A,B) and anxiety-like behaviours (Supplementary Fig. S1C) as compared to age-matched WT littermates, arguing against potential confounding behavioural interpretation as ADHD symptoms have been reported to decrease with age in both humans^47^ and animal models^48^. Moreover, the evidence that the behaviour of KO mice can be ameliorated by the mood stabilizer valproate speaks in favour of a manic-like phenotype.

Increased sucrose consumption observed in some animal models of mania has been proposed to recapitulate the hyper-hedonic behaviour displayed by BD patients^29^. We could not detect any difference between WT and KO animals in this behavioural paradigm. However, only 40% of BD patients show comorbidity with substance abuse disorder, arguing for the diversity of symptoms among BD patients^49^ and therefore for the complexity of BD^38^. The condition observed in *Tph2* mutant mice is likely due to serotonin depletion *per se* as behavioural hyperactivity has been observed in mice in which *Tph2* was targeted in adulthood^23^. Therefore, a primary role of 5-HT depletion is likely to be the main causative effect of the phenotypes observed here, although a role of abnormal developmental trajectories caused by 5-HT depletion cannot be entirely ruled out. Supporting a developmental origin of these changes, several linkage studies have described an association between bipolar disorder and single nucleotide polymorphisms in genes such as TPH2, HTR1A, HTR2A, HTR2C or SLC6A4^50–55^. This suggests the existence of an interplay between developmental and neuroadaptive mechanism caused by aberrant 5-HT neurotransmission in the etiology of BD.

Guided by fMRI mapping, we identified the hippocampus as a key substrate for the behavioural changes observed. Strikingly, the transcriptional changes in the hippocampus of KO mice showed remarkable similarity to those observed in neurons derived from bipolar disorder patients^13^. Moreover, these transcriptional changes underlie a common hyperactive phenotype in the hippocampus of KO mice and in BD-derived hippocampal-like neurons, which is in keeping with an abnormal neuroplasticity and a reduced inhibitory drive identified in the hippocampus of preclinical models and bipolar disorder patients^14,15,56,57^. Owing to these similarities we propose that the hyperactivity in *Tph2* mutants is phenotypically and mechanistically relevant for BD associated manic states.

Emotional instability and exaggerated fluctuations in mood are the distinctive traits in bipolar disorder. Similarly to BD patients who experience abnormal mood transitions from the manic to the depressive state^38,58,59^, *Tph2* mutant mice switch toward depressive-like behaviours upon exposure to stressful environment. The evidence that in *Tph2* knockout mice a relatively small number of genes is reversed with stress indicates the existence of two distinct transcriptional signatures that are associated to either manic- or depressive-like behaviours, thus suggesting that hippocampal hyperactivity is mood-phase dependent.

Dysregulated adaptive mechanisms to stress may be relevant for mood shifts. Accordingly, the kindling hypothesis posits that stressful events trigger the onset of affective episode and their recurrence overburdens adaptive mechanisms to stress, thus resulting in a gradual process of sensitization^38,60^. Even though WT-S mice displayed an increased immobility in the FST and TST, their transcriptional signature 24 hours after the last stress session clearly showed the establishment of molecular changes increasing neuroplasticity that are likely involved in the adaptation to stress. According to this view, the strong transcriptional similarity between KO and WT-S mice suggests that adaptive mechanisms to stress are constitutively active in the hippocampus of KO mice housed in standard conditions. Our evidence that recurrence of stressful episodes in KO-S reverts the transcriptional signature including the expression level of BDNF and TrkB demonstrates that the appropriate activation of neuroplasticity adaptive mechanisms to environmental insults is precluded. Such a blunted response is indicative of a possible overburden of these mechanisms. Somewhat surprisingly, when augmented levels of serotonin were induced, higher vulnerability to adverse living environment was described in a recent study by Alboni and collaborators, who reported a stress-induced worsening of hippocampal neuroplasticity and depressive-like behaviour in fluoxetine-administered mice^61^. Taken together, these data support the hypothesis that physiological 5-HT levels are critical for an organism to adapt to the living environment and cope with stress.

Interestingly, contradictory findings regarding the assessment of depression-like behavior of *Tph2* knock-out mice have been generated^62–64^. Indeed, both reduced and increased immobility have been observed in the FST, whereas no difference has been observed in the TST. Moreover, while Mosienko and colleagues described a decreased anxiety in these animals, other groups did not observe any change in anxiety levels. We believe these controversial results could be further indicators of an unstable and dysphoric behavioural condition that is likely unmasked by subtle differences in environmental conditions present in distinct laboratories rather than to phenotype penetrance and/or genetic background variability.

Collectively, our findings reveal that impaired 5-HT neurotransmission is a critical determinant of maladaptive behaviour and aberrant hippocampal neuroplasticity relevant for bipolar disorder, thus supporting the use of *Tph2*−/− mice as a new research tool for mechanistic and therapeutic research in bipolar disorder.

## Electronic supplementary material

Supplementary Information
