# Supplementary material for: Serotonin depletion causes valproate-responsive manic-like condition and increased hippocampal neuroplasticity that are reversed by stress
Source: Sci Rep. 2018 Aug 7;8:11847. doi: 10.1038/s41598-018-30291-2 (PMC6081464; doi:10.1038/s41598-018-30291-2)
Supplement: Supplementary file 1 — Supplementary Information [file 41598_2018_30291_MOESM1_ESM.pdf]

# Serotonin depletion causes valproate-responsive manic-like condition and increased hippocampal neuroplasticity that are reversed by stress

Giacomo Maddaloni, Sara Migliarini, Francesco Napolitano, Andrea Giorgi, Serena Nazzi, Daniele Biasci, Alessia De Felice, Marta Gritti, Anna Cavaccini, Alberto Galbusera, Sara Franceschi, Francesca Lessi, Marco La Ferla, Paolo Aretini, Chiara Maria Mazzanti, Raffaella Tonini, Alessandro Gozzi, Alessandro Usiello & Massimo Pasqualetti

**SUPPLEMENTARY INFORMATIONS**

**Supplementary figures and legends**

**Supplementary Figure S1**

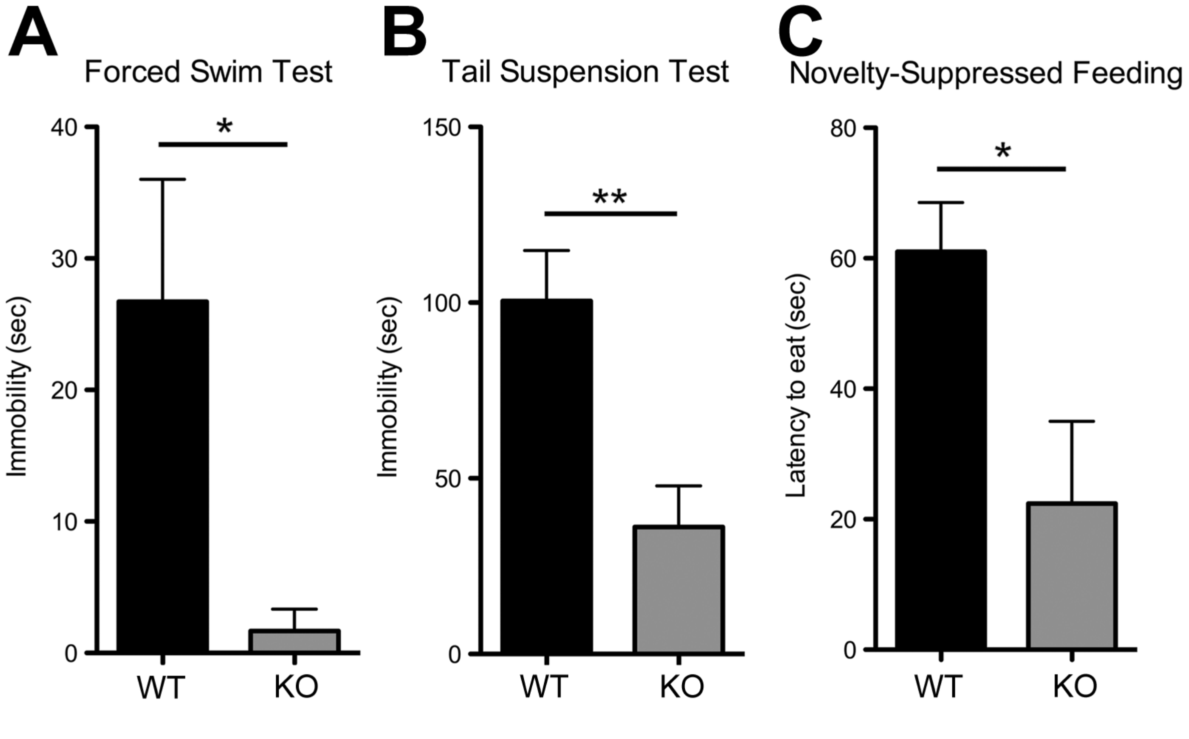


**Supplementary Figure S1:** Behavioural analysis in aged animals. (**A**) Immobility time in the FST and (**B**) TST. (**C**) Latency to eat in the NSF. Data are expressed as mean ± s.e.m., *p < 0.05, **p < 0.01.

**Supplementary Figure S2**

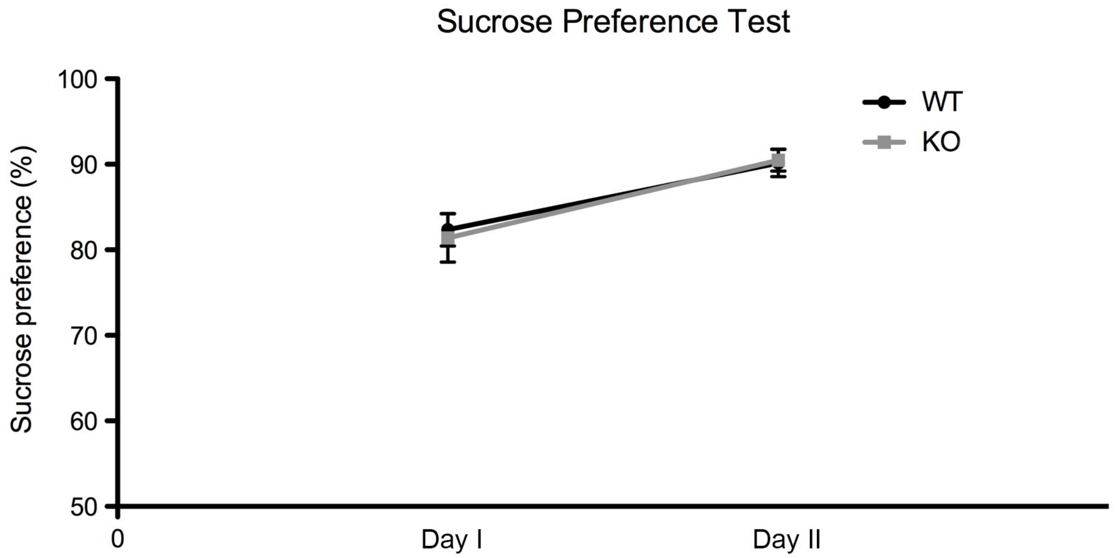


**Supplementary Figure S2:** Hedonic behaviour in *Tph2* KO mice. Results showed no difference in sucrose preference (calculated as percentage of sucrose intake over the total volume of fluid intake) between WT and KO mice both on day I and day II. Moreover, both WT and KO mice showed increased preference on day II. Data are expressed as mean ± s.e.m.

**Supplementary Figure S3**

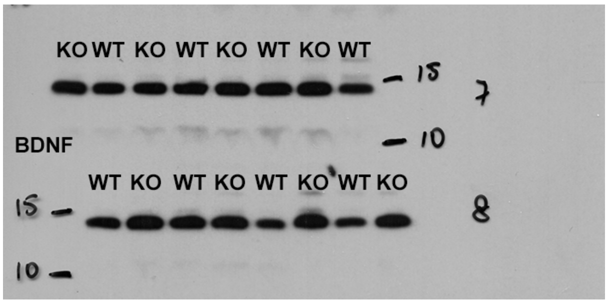


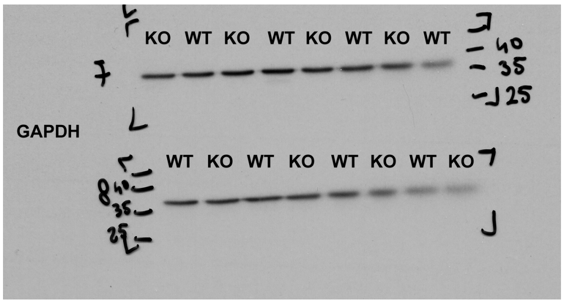


**Supplementary Figure S3:** Full length blots showing BDNF protein levels supporting figure 2C.

**Supplementary Figure S4**

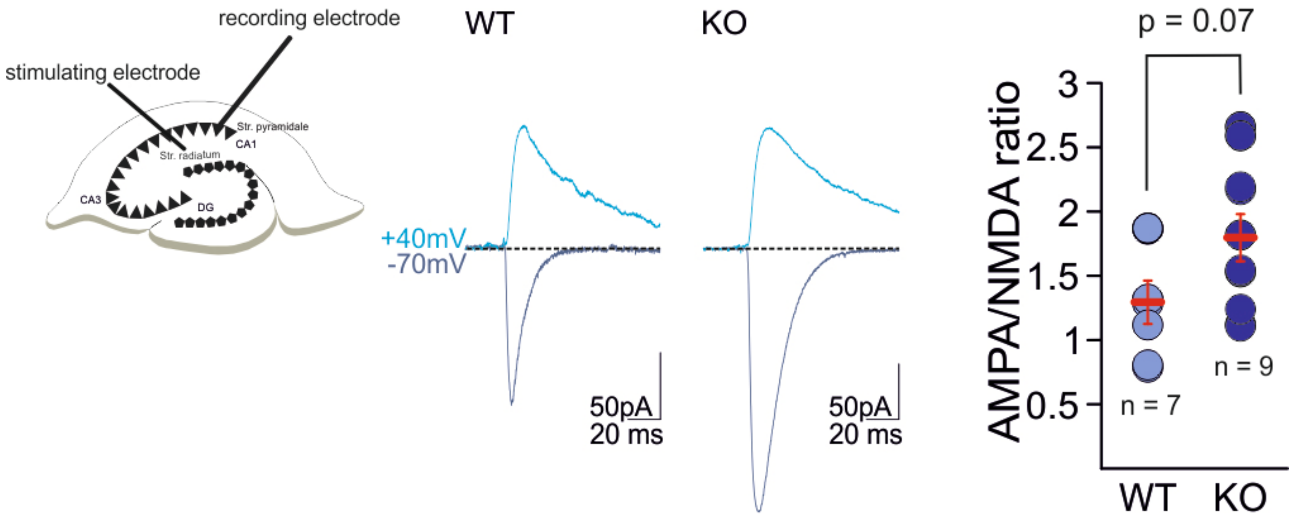


**Supplementary Figure S4:** AMPA to NMDA ratio in CA1 hippocampal neurons. (left) Schematic of recording configuration; (middle) Representative traces form whole-cell voltage-clamp experiments showing AMPA receptor- and NMDA-receptor mediated currents in CA1 pyramidal neurons of WT and KO mutant mice; (right) AMPA to NMDA ratio in the two mouse cohorts.

**Supplementary Figure S5**

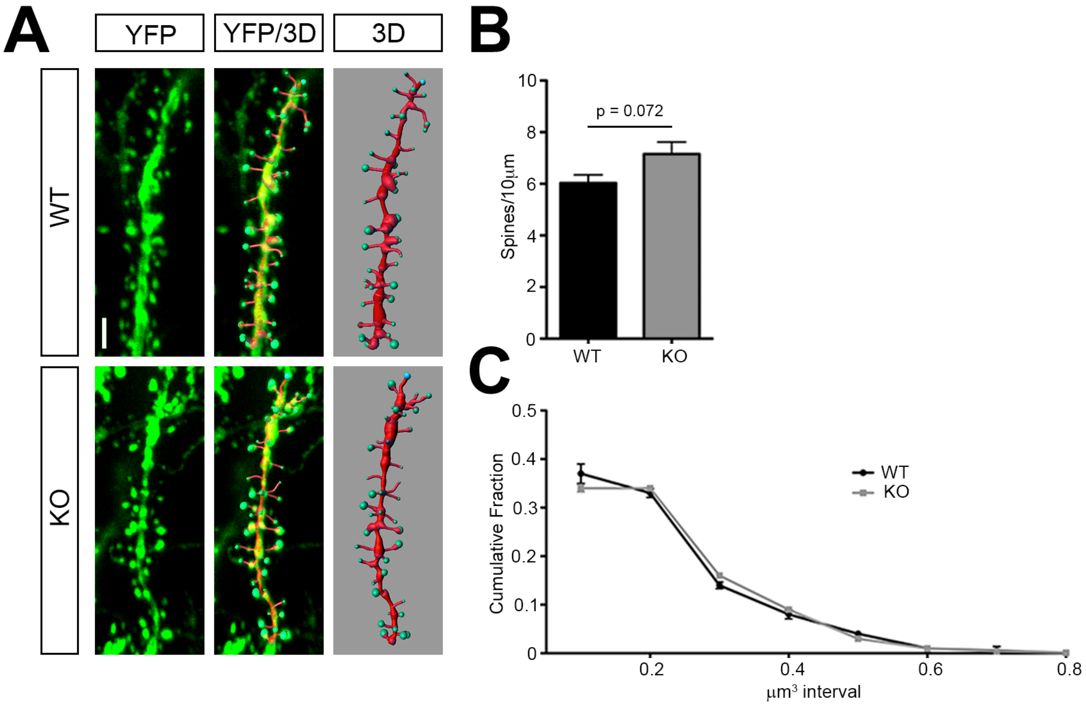


**Supplementary Figure S5:** Dendritic spine analysis in CA1 apical dendrites. (**A**) Representative images of YFP-labelled CA1 apical dendrites and their 3D reconstruction. (**B**, **C**) Quantification of spine density (WT n = 7; KO n = 8, for spine density one-way ANOVA, $F_{1,13} = 3.624$, $p = 0.072$) and volume distribution. Data are expressed as mean ± s.e.m. n indicates biological replicates. Scale bar = 2μm.

**Supplementary Figure S6**

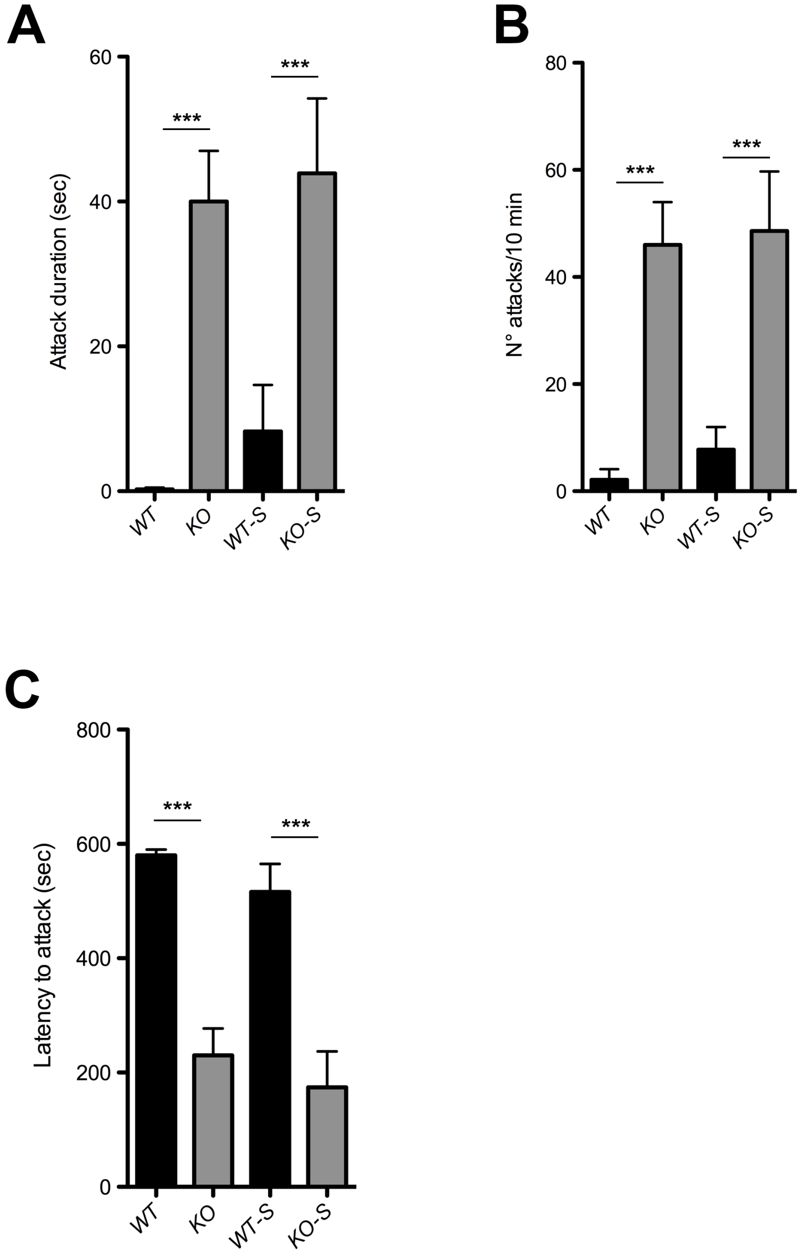


**Supplementary Figure S6:** Aggressive behaviour characterization in uCMS exposed mice. (**A**) Attack duration (n = 12; two-way ANOVA followed by Fisher's post-hoc test, effect of genotype $F_{1,44} = 27.073$, $p < 0.0001$; KO vs WT $p < 0.0001$, KO-S vs WT-S $p < 0.0001$) (**B**) latency to the first attack (n = 12; two-way ANOVA followed by Fisher's post-hoc test, effect of genotype $F_{1,44} = 27.127$, $p < 0.0001$; KO vs WT $p < 0.0001$, KO-S vs WT-S $p < 0.0001$) and (**C**) attack frequency ( n = 12; two-way ANOVA followed by Fisher's post-hoc test, effect of genotype $F_{1,44} = 58.592$, $p < 0.0001$; KO vs WT $p < 0.0001$, KO-S vs WT-S $p < 0.0001$) in the NAAT. Data are expressed as mean ± s.e.m. n indicates biological replicates. *$p < 0.05$, **$p < 0.01$, ***$p < 0.001$. NAAT, Neutral Arena Aggression Test.

**Supplementary Figure S7**

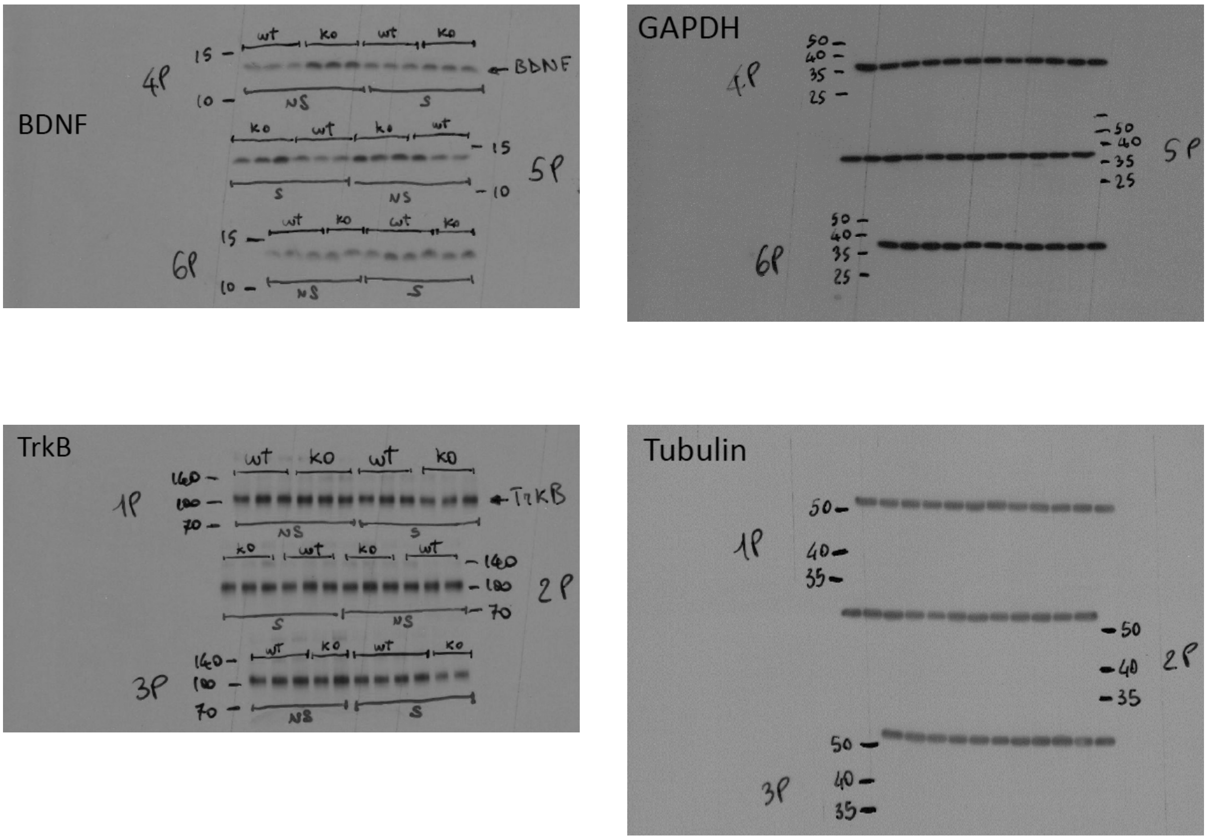


**Supplementary Figure S7.** Full length blots showing BDNF and TrkB protein levels supporting figure 4C and 4D, respectively. NS = non-stress group; S = stress group.

## Supplementary tables

### **Supplementary Table S1:** Biological process Gene Ontology categories for KO/WT comparison

| Biological process | p-value | Biological process | p-value |
|---|---|---|---|
| single-multicellular organism process (GO:0044707) | 0,000000 | anion transmembrane transport (GO:0098656) | 0,001550 |
| ion transport (GO:0006811) | 0,000000 | localization (GO:0051179) | 0,001930 |
| anion transport (GO:0006820) | 0,000000 | neurogenesis (GO:0022008) | 0,002450 |
| multicellular organism development (GO:0007275) | 0,000000 | positive regulation of metabolic process (GO:0009893) | 0,002870 |
| single-organism developmental process (GO:0044767) | 0,000000 | single-organism cellular process (GO:0044763) | 0,003620 |
| developmental process (GO:0032502) | 0,000000 | pattern specification process (GO:0007389) | 0,004920 |
| multicellular organismal process (GO:0032501) | 0,000000 | regulation of biological process (GO:0050789) | 0,005490 |
| anatomical structure development (GO:0048856) | 0,000000 | nervous system development (GO:0007399) | 0,005800 |
| single-organism process (GO:0044699) | 0,000001 | regulation of cell proliferation (GO:0042127) | 0,005940 |
| system development (GO:0048731) | 0,000008 | regulation of localization (GO:0032879) | 0,010200 |
| single-organism transport (GO:0044765) | 0,000009 | regulation of response to stimulus (GO:0048583) | 0,010600 |
| ion transmembrane transport (GO:0034220) | 0,000011 | regulation of multicellular organismal development (GO:2000026) | 0,010800 |
| single-organism localization (GO:1902578) | 0,000022 | regulation of cellular process (GO:0050794) | 0,011100 |
| biological regulation (GO:0065007) | 0,000082 | nitrogen compound transport (GO:0071705) | 0,011800 |
| sensory organ development (GO:0007423) | 0,000129 | eye development (GO:0001654) | 0,013500 |
| positive regulation of biological process (GO:0048518) | 0,000181 | regulation of biological quality (GO:0065008) | 0,013600 |
| transport (GO:0006810) | 0,000192 | muscle contraction (GO:0006936) | 0,013800 |
| animal organ development (GO:0048513) | 0,000204 | amino acid transport (GO:0006865) | 0,015300 |
| anatomical structure morphogenesis (GO:0009653) | 0,000298 | cell development (GO:0048468) | 0,015700 |
| transmembrane transport (GO:0055085) | 0,000340 | regulation of metabolic process (GO:0019222) | 0,016900 |
| cell differentiation (GO:0030154) | 0,000569 | response to organic substance (GO:0010033) | 0,019600 |
| organic anion transport (GO:0015711) | 0,000590 | positive regulation of cellular process (GO:0048522) | 0,021000 |
| establishment of localization (GO:0051234) | 0,000610 | dicarboxylic acid transport (GO:0006835) | 0,022600 |
| regulation of multicellular organismal process (GO:0051239) | 0,000714 | neuron development (GO:0048666) | 0,025700 |
| generation of neurons (GO:0048699) | 0,000763 | regulation of transport (GO:0051049) | 0,025900 |
| cellular process (GO:0009987) | 0,000841 | renal system process (GO:0003014) | 0,027500 |
| cellular developmental process (GO:0048869) | 0,000909 | neuron projection development (GO:0031175) | 0,038000 |

| | | | |
|---|---|---|---|
| carboxylic acid transport (GO:0046942) | 0,000973 | antigen processing and presentation of peptide antigen via MHC class I (GO:0002474) | 0,038500 |
| organic acid transport (GO:0015849) | 0,001110 | positive regulation of transport (GO:0051050) | 0,038600 |
| response to external stimulus (GO:0009605) | 0,001230 | cellular response to external stimulus (GO:0071496) | 0,039300 |
| | | positive regulation of nitrogen compound metabolic process (GO:0051173) | 0,044000 |

**Supplementary Table S2:** Cellular component Gene Ontology categories for KO/WT comparison

| Cellular Component | p-value | Cellular Component | p-value |
|---|---|---|---|
| plasma membrane part (GO:0044459) | 0,000000 | cell surface (GO:0009986) | 0,004330 |
| cell periphery (GO:0071944) | 0,000002 | membrane part (GO:0044425) | 0,005220 |
| vesicle (GO:0031982) | 0,000002 | membrane protein complex (GO:0098796) | 0,006040 |
| plasma membrane (GO:0005886) | 0,000003 | extracellular exosome (GO:0070062) | 0,006070 |
| membrane region (GO:0098589) | 0,000005 | cell part (GO:0044464) | 0,006070 |
| whole membrane (GO:0098805) | 0,000007 | cell (GO:0005623) | 0,006390 |
| plasma membrane region (GO:0098590) | 0,000016 | extracellular vesicle (GO:1903561) | 0,006720 |
| extracellular region part (GO:0044421) | 0,000450 | ion channel complex (GO:0034702) | 0,007970 |
| neuron projection (GO:0043005) | 0,000704 | endoplasmic reticulum lumen (GO:0005788) | 0,009700 |
| intrinsic component of plasma membrane (GO:0031226) | 0,000884 | MHC protein complex (GO:0042611) | 0,012300 |
| endoplasmic reticulum part (GO:0044432) | 0,001230 | endoplasmic reticulum (GO:0005783) | 0,013600 |
| cell projection (GO:0042995) | 0,001400 | transmembrane transporter complex (GO:1902495) | 0,017500 |
| MHC class I protein complex (GO:0042612) | 0,001430 | transporter complex (GO:1990351) | 0,020600 |
| endoplasmic reticulum exit site (GO:0070971) | 0,001430 | organelle (GO:0043226) | 0,022600 |
| integral component of plasma membrane (GO:0005887) | 0,001480 | smooth endoplasmic reticulum (GO:0005790) | 0,024500 |
| neuron part (GO:0097458) | 0,002100 | plasma membrane protein complex (GO:0098797) | 0,025600 |
| extracellular organelle (GO:0043230) | 0,002340 | endomembrane system (GO:0012505) | 0,033300 |
| extracellular region (GO:0005576) | 0,002580 | endoplasmic reticulum chaperone complex (GO:0034663) | 0,040800 |
| Golgi medial cisterna (GO:0005797) | 0,002920 | cell projection membrane (GO:0031253) | 0,044600 |

**Supplementary Table S3:** Molecular functions Gene Ontology categories for KO/WT comparison

| Molecular function | p-value | Molecular function | p-value |
|---|---|---|---|
| protein binding (GO:0005515) | 0,000002 | beta-2-microglobulin binding (GO:0030881) | 0,002020 |
| binding (GO:0005488) | 0,000004 | T cell receptor binding (GO:0042608) | 0,002710 |
| ion transmembrane transporter activity (GO:0015075) | 0,000011 | unfolded protein binding (GO:0051082) | 0,004240 |
| substrate-specific transmembrane transporter activity (GO:0022891) | 0,000037 | sodium ion transmembrane transporter activity (GO:0015081) | 0,010900 |
| anion transmembrane transporter activity (GO:0008509) | 0,000054 | metal ion transmembrane transporter activity (GO:0046873) | 0,015700 |
| transporter activity (GO:0005215) | 0,000063 | ion channel activity (GO:0005216) | 0,016000 |
| substrate-specific transporter activity (GO:0022892) | 0,000116 | substrate-specific channel activity (GO:0022838) | 0,020700 |
| transmembrane transporter activity (GO:0022857) | 0,000156 | cation transmembrane transporter activity (GO:0008324) | 0,025800 |
| gated channel activity (GO:0022836) | 0,001130 | monovalent inorganic cation transmembrane transporter activity (GO:0015077) | 0,028400 |
| TAP binding (GO:0046977) | 0,001470 | passive transmembrane transporter activity (GO:0022803) | 0,039100 |
| protein dimerization activity (GO:0046983) | 0,001560 | channel activity (GO:0015267) | 0,039100 |

**Supplementary Table S4:** Biological process Gene Ontology categories for KO-S/KO comparison

| Biological Process | p-value | Biological Process | p-value |
|---|---|---|---|
| ion transport (GO:0006811) | 0,000007 | multicellular organism development (GO:0007275) | 0,006060 |
| single-multicellular organism process (GO:0044707) | 0,000027 | transport (GO:0006810) | 0,006500 |
| single-organism transport (GO:0044765) | 0,000259 | cellular component organization (GO:0016043) | 0,008000 |
| animal organ development (GO:0048513) | 0,000477 | cellular component organization or biogenesis (GO:0071840) | 0,008710 |
| single-organism developmental process (GO:0044767) | 0,000584 | positive regulation of biological process (GO:0048518) | 0,009230 |
| single-organism process (GO:0044699) | 0,000618 | anion transport (GO:0006820) | 0,010300 |
| single-organism localization (GO:1902578) | 0,000656 | blood circulation (GO:0008015) | 0,011500 |
| developmental process (GO:0032502) | 0,000729 | regulation of biological quality (GO:0065008) | 0,011800 |
| ion transmembrane transport (GO:0034220) | 0,000749 | circulatory system process (GO:0003013) | 0,012900 |
| extracellular matrix organization (GO:0030198) | 0,001780 | system development (GO:0048731) | 0,013200 |
| extracellular structure organization (GO:0043062) | 0,001870 | establishment of localization (GO:0051234) | 0,019100 |
| multicellular organismal process (GO:0032501) | 0,002190 | single-organism cellular process (GO:0044763) | 0,021800 |
| localization (GO:0051179) | 0,002260 | positive regulation of cellular process (GO:0048522) | 0,025100 |
| anatomical structure morphogenesis (GO:0009653) | 0,003590 | anatomical structure development (GO:0048856) | 0,032400 |
| response to chemical (GO:0042221) | 0,003880 | response to inorganic substance (GO:0010035) | 0,041400 |
| transmembrane transport (GO:0055085) | 0,003910 | cellular process (GO:0009987) | 0,049500 |

**Supplementary Table S5:** Cellular component Gene Ontology categories for KO-S/KO comparison

| Cellular Component | p-value | Cellular Component | p-value |
|---|---|---|---|
| membrane-bounded organelle (GO:0043227) | 0,000008 | extracellular organelle (GO:0043230) | 0,003230 |
| organelle (GO:0043226) | 0,000015 | cell periphery (GO:0071944) | 0,005020 |
| extracellular matrix (GO:0031012) | 0,000131 | plasma membrane (GO:0005886) | 0,005750 |
| extracellular region part (GO:0044421) | 0,000259 | extracellular region (GO:0005576) | 0,005770 |
| cell surface (GO:0009986) | 0,000391 | extracellular exosome (GO:0070062) | 0,007680 |
| vesicle (GO:0031982) | 0,001060 | cell part (GO:0044464) | 0,010300 |
| endoplasmic reticulum chaperone complex (GO:0034663) | 0,001080 | cell (GO:0005623) | 0,010900 |
| membrane region (GO:0098589) | 0,001530 | plasma membrane region (GO:0098590) | 0,016900 |
| plasma membrane part (GO:0044459) | 0,001810 | basolateral plasma membrane (GO:0016323) | 0,035600 |
| whole membrane (GO:0098805) | 0,002610 | basement membrane (GO:0005604) | 0,041800 |
| extracellular vesicle (GO:1903561) | 0,003070 | | |

**Supplementary Table S6:** Molecular function Gene Ontology categories for KO-S/KO comparison

| **Molecular Function** | **p-value** |
|---|---|
| binding (GO:0005488) | 0,000000 |
| transporter activity (GO:0005215) | 0,000276 |
| ion transmembrane transporter activity (GO:0015075) | 0,000739 |
| substrate-specific transporter activity (GO:0022892) | 0,001640 |
| transmembrane transporter activity (GO:0022857) | 0,001970 |
| substrate-specific transmembrane activity (GO:0022891) | 0,002230 |
| protein binding (GO:0005515) | 0,003300 |
| metal ion transmembrane transporter activity (GO:0046873) | 0,011500 |
| cation transmembrane transporter activity (GO:0008324) | 0,028800 |

**Supplementary Table S7:** Biological process Gene Ontology categories for WT-S/WT comparison

| Biological process | p-value | Biological process | p-value |
|---|---|---|---|
| anatomical structure development (GO:0048856) | 0,00000000 | regulation of hormone levels (GO:0010817) | 0,00048100 |
| developmental process (GO:0032502) | 0,00000000 | establishment of localization (GO:0051234) | 0,00058000 |
| positive regulation of multicellular organismal process (GO:0051240) | 0,00000000 | cation transport (GO:0006812) | 0,00061800 |
| cellular process (GO:0009987) | 0,00000006 | response to endogenous stimulus (GO:0009719) | 0,00064900 |
| collagen fibril organization (GO:0030199) | 0,00000009 | muscle system process (GO:0003012) | 0,00066800 |
| extracellular matrix organization (GO:0030198) | 0,00000012 | organic acid transport (GO:0015849) | 0,00104000 |
| anion transmembrane transport (GO:0098656) | 0,00000020 | ion transport (GO:0006811) | 0,00116000 |
| response to organonitrogen compound (GO:0010243) | 0,00000024 | cellular component organization or biogenesis (GO:0071840) | 0,00169000 |
| connective tissue development (GO:0061448) | 0,00000043 | organic anion transport (GO:0015711) | 0,00205000 |
| single-organism developmental process (GO:0044767) | 0,00000067 | anatomical structure morphogenesis (GO:0009653) | 0,00213000 |
| receptor protein serine/threonine kinase signaling pathway (GO:0090092) | 0,00000079 | single-organism process (GO:0044699) | 0,00215000 |
| system process (GO:0003008) | 0,00000181 | regulation of cell differentiation (GO:0045595) | 0,00224000 |
| cellular component organization (GO:0016043) | 0,00000236 | circulatory system process (GO:0003013) | 0,00229000 |
| positive regulation of developmental process (GO:0051094) | 0,00000256 | hormone transport (GO:0009914) | 0,00335000 |
| cell-cell signaling (GO:0007267) | 0,00000419 | single-multicellular organism process (GO:0044707) | 0,00362000 |
| nitrogen compound transport (GO:0071705) | 0,00002190 | tissue development (GO:0009888) | 0,00405000 |
| localization (GO:0051179) | 0,00003320 | regulation of developmental process (GO:0050793) | 0,00518000 |
| blood circulation (GO:0008015) | 0,00003380 | response to chemical (GO:0042221) | 0,00519000 |
| organ morphogenesis (GO:0009887) | 0,00004250 | response to nitrogen compound (GO:1901698) | 0,00763000 |
| single-organism transport (GO:0044765) | 0,00004510 | anion transport (GO:0006820) | 0,00986000 |
| transmembrane transport (GO:0055085) | 0,00005260 | regulation of biological quality (GO:0065008) | 0,01160000 |
| animal organ development (GO:0048513) | 0,00005590 | metal ion transport (GO:0030001) | 0,01220000 |
| response to organic substance (GO:0010033) | 0,00006270 | amino acid transport (GO:0006865) | 0,01350000 |
| cell differentiation (GO:0030154) | 0,00008380 | regulation of multicellular organismal development (GO:2000026) | 0,01360000 |
| carboxylic acid transport (GO:0046942) | 0,00010700 | cellular developmental process (GO:0048869) | 0,02140000 |
| sensory organ development (GO:0007423) | 0,00013000 | single-organism localization (GO:1902578) | 0,02400000 |
| extracellular structure organization (GO:0043062) | 0,00024000 | single-organism cellular process (GO:0044763) | 0,02570000 |
| system development (GO:0048731) | 0,00024300 | transport (GO:0006810) | 0,02700000 |
| multicellular organism development (GO:0007275) | 0,00027500 | ion transmembrane transport (GO:0034220) | 0,03050000 |

| | | | |
|---|---|---|---|
| muscle contraction (GO:0006936) | 0,00031500 | neurotransmitter transport (GO:0006836) | 0,03630000 |
| | | regulation of multicellular organismal process (GO:0051239) | 0,03740000 |

**Supplementary Table S8:** Cellular component Gene Ontology categories for WT-S/WT comparison

| **Cellular Component** | **p-value** | **Cellular Component** | **p-value** |
|---|---|---|---|
| vesicle (GO:0031982) | 0,00000077 | extracellular vesicle (GO:1903561) | 0,00444000 |
| | | extracellular organelle (GO:0043230) | 0,00468000 |
| collagen trimer (GO:0005581) | 0,00000293 | integral component of plasma membrane (GO:0005887) | 0,00571000 |
| extracellular matrix component (GO:0044420) | 0,00000974 | proteinaceous extracellular matrix (GO:0005578) | 0,00816000 |
| extracellular space (GO:0005615) | 0,00004060 | extracellular exosome (GO:0070062) | 0,01090000 |
| extracellular region part (GO:0044421) | 0,00005290 | plasma membrane part (GO:0044459) | 0,02080000 |
| complex of collagen trimers (GO:0098644) | 0,00015000 | cytoplasmic vesicle membrane (GO:0030659) | 0,02160000 |
| extracellular matrix (GO:0031012) | 0,00015900 | basal part of cell (GO:0045178) | 0,03060000 |
| extracellular region (GO:0005576) | 0,00057400 | whole membrane (GO:0098805) | 0,03380000 |
| fibrillar collagen trimer (GO:0005583) | 0,00115000 | cytoplasmic vesicle part (GO:0044433) | 0,03590000 |
| banded collagen fibril (GO:0098643) | 0,0011000 | membrane-bounded organelle (GO:0043227) | 0,04090000 |
| organelle (GO:0043226) | 0,00290000 | cell periphery (GO:0071944) | 0,04370000 |
| intrinsic component of plasma membrane (GO:0031226) | 0,00392000 | plasma membrane (GO:0005886) | 0,04950000 |

**Supplementary Table S9:** Molecular Function Gene Ontology categories for WT-S/WT comparison

| Molecular Function | p-value | Molecular function | p-value |
|---|---|---|---|
| transporter activity (GO:0005215) | 0,0000000 | growth factor binding (GO:0019838) | 0,0037400 |
| substrate-specific transporter activity (GO:0022892) | 0,0000051 | ion binding (GO:0043167) | 0,0050100 |
| transmembrane transporter activity (GO:0022857) | 0,0000054 | cation transmembrane transporter activity (GO:0008324) | 0,0071600 |
| ion transmembrane transporter activity (GO:0015075) | 0,0000072 | metal ion transmembrane transporter activity (GO:0046873) | 0,0135000 |
| substrate-specific transmembrane transporter activity (GO:0022891) | 0,0000270 | monovalent inorganic cation transmembrane transporter activity (GO:0015077) | 0,0203000 |
| anion transmembrane transporter activity (GO:0008509) | 0,0000396 | active ion transmembrane transporter activity (GO:0022853) | 0,0222000 |
| binding (GO:0005488) | 0,0002460 | inorganic cation transmembrane transporter activity (GO:0022890) | 0,0251000 |
| symporter activity (GO:0015293) | 0,0005840 | sodium ion transmembrane transporter activity (GO:0015081) | 0,0324000 |
| secondary active transmembrane transporter activity (GO:0015291) | 0,0006650 | amino acid transmembrane transporter activity (GO:0015171) | 0,0342000 |
| active transmembrane transporter activity (GO:0022804) | 0,0008350 | organic anion transmembrane transporter activity (GO:0008514) | 0,0351000 |
| anion:cation symporter activity (GO:0015296) | 0,0019100 | transcription factor activity, RNA polymerase II proximal region binding (GO:0000982) | 0,0371000 |
| platelet-derived growth factor binding (GO:0048407) | 0,0028600 | | |
